# Risk management-based security evaluation model for telemedicine systems

**DOI:** 10.1186/s12911-020-01145-7

**Published:** 2020-06-10

**Authors:** Dong-won Kim, Jin-young Choi, Keun-hee Han

**Affiliations:** Information Security Department, Korea University, Seoul, Republic of Korea

**Keywords:** Telemedicine security, Medical information security, Smart medical security, Telecare security

## Abstract

**Background:**

Infectious diseases that can cause epidemics, such as COVID-19, SARS-CoV, and MERS-CoV, constitute a major social issue, with healthcare providers fearing secondary, tertiary, and even quaternary infections. To alleviate this problem, telemedicine is increasingly being viewed as an effective means through which patients can be diagnosed and medications prescribed by doctors via untact Thus, concomitant with developments in information and communication technology (ICT), medical institutions have actively analyzed and applied ICT to medical systems to provide optimal medical services. However, with the convergence of these diverse technologies, various risks and security threats have emerged. To protect patients and improve telemedicine quality for patient safety, it is necessary to analyze these risks and security threats comprehensively and institute appropriate countermeasures.

**Methods:**

The security threats likely to be encountered in each of seven telemedicine service areas were analyzed, and related data were collected directly through on-site surveys by a medical institution. Subsequently, an attack tree, the most popular reliability and risk modeling approach for systematically characterizing the potential risks of telemedicine systems, was examined and utilized with the attack occurrence probability and attack success probability as variables to provide a comprehensive risk assessment method.

**Results:**

In this study, the most popular modelling method, an attack tree, was applied to the telemedicine environment, and the security concerns for telemedicine systems were found to be very large. Risk management and evaluation methods suitable for the telemedicine environment were identified, and their benefits and potential limitations were assessed.

**Conclusion:**

This research should be beneficial to security experts who wish to investigate the impacts of cybersecurity threats on remote healthcare and researchers who wish to identify new modeling opportunities to apply security risk modeling techniques.

## Background

Healthcare is evolving towards preventive medical services for lifelong personal health management [1]. Concomitant with the fusion of healthcare with information and communication technology (ICT), various new services and networked medical devices have been developed. These networked devices provide services such as telemedicine, health information exchange, and precision medicine. As these devices have immediate effects on the lives of patients, security management is critical [2–12]. In particular, data transmission from wired to wireless networks requires specific security guidelines for data processing and management and medical device development [13].

In addition, infectious diseases such as COVID-19 [14, 15], SARS-CoV [16], and MERS-CoV [17] cause major social problems and are known to result in severe respiratory or gastrointestinal complications when they infect animals or people. Coronavirus (CoV) was previously considered to be a pathogen that causes minor symptoms in the community in the form of endemic infection, but there is a growing need to introduce telemedicine that can be utilized to diagnose and prescribe appropriate medication owing to the growing fear of secondary and tertiary infections [15].

Many recently developed medical devices are upgradable, which further increases the potential security threats that can affect them. For example, the vulnerability of insulin pumps to hacking was reported both in 2010 and 2013 [18]. Additionally, in August 2016, an intensive care unit infusion pump sensor without communication functionality was hacked using a low-cost infrared laser [19].

Telemedicine can be broadly categorized into five types: ① videoconference-based patient consultations using the Picture Archiving Communications System in large hospitals, ② multimedia transmission to provide remote services such as first-aid directions, ③ remote home care, ④ remote training of patients or health professionals, and ⑤ online medical counseling and health information sharing [20].

With recent advances in internet of things technology, connectivity between objects is being driven by the medical/electronic sector [21, 22]. Healthcare services value prevention and management over the treatment of future diseases, which can be extended to diagnosis, surgery, and treatment [23]. The healthcare field is being labeled as the “next big thing,” and innovative developments are highly anticipated [24–26]. Implantable medical devices (IMDs), which monitor patient health and heal affected body parts, are vital in healthcare [27]. Examples of IMDs include cardiac pacemakers and defibrillators, which monitor and treat heart conditions; deep brain simulators, which treat epilepsy or Parkinson’s disease; drug delivery systems in the form of infusion pumps; and bio-instruments that acquire and process bio-signals [28].

However, IMDs, which are equipped with advanced computing and communications capabilities, also entail security and privacy threats. In some cases, such threats can have fatal consequences. Deliberate attacks can result in death if they cause intentional malfunctions, and intentional attacks can be considerably more difficult to detect than accidental attacks [29]. IMDs also store and transmit highly sensitive medical information that should be protected under the laws of Europe (e.g., Directive 95/46/ECC) and the United States (e.g., CFR 164.312) [30, 31]. Experiments have demonstrated how treatment functions can be disabled or reprogrammed to induce shock conditions in patients through wireless connections, as a part of an attack on an IMD [32–34]. Moreover, the device can be sabotaged by intentionally discharging the battery. In such cases, it is often necessary to replace the IMD through surgery. For cardiac IMDs, the power can be switched off using a magnetic field [35], which led to former U.S. Vice President Dick Cheney disabling the Wi-Fi function of his implantable cardioverter–defibrillator to prevent remote assassination attempts [2].

Security requirements pertaining to the processing and management of large amounts of data transmitted wirelessly are essential, and the importance of cybersecurity in the development of medical devices is growing [3]. Various medical devices that have evolved in recent years have had several functional advances, but the potential security threats have also continued to grow. The possibility of hacking of medical devices has already been reported in several articles [4, 6], and research has demonstrated the possibility of healthcare-related security accidents.

A common paradigm in the performance of cyber risk assessment is to form two adversarial teams consisting of a “red team” whose job is to think like an attacker and a “blue team” that seeks to defend the system by developing countermeasures [36]. In many situations, red team information is applied to model the systems using techniques such as attack trees [10], attack-defense trees [37], event trees [38, 39], Markov models [40], decision diagrams such as binary decision diagrams [41], and fault trees [42, 43].

The “attack tree” process [10] is a systematic method for determining the characteristics of system security based on all attacks to which a system is exposed [6–9]. Identifying all possible defined attacks facilitates analysis of all possible cyberattack access paths and selection of the best-suited countermeasures and their optimal deployment. An attack tree consists of nodes, edges, and connectors, with each node corresponding to an attack step. The root node represents the ultimate goal of the attacker, while the children of a given node represent the subgoals. The edges represent the state change caused by the actions of the attacker. A connector is a gate (either OR (disjunctive) or AND (conjunctive)) for the nodes with two or more children for advancement to reach the attack goal [10].

In this study, the most popular modeling approach, an attack tree, was utilized, with the attack occurrence probability (AOP) and attack success probability (ASP) as variables, to develop a risk assessment method, and the benefits and potential limitations of this method were assessed.

The remainder of this paper is organized as follows. Section II describes the telemedicine system architecture and discusses potential security threats and scenarios that may arise therefrom. Section III outlines the proposed risk assessment method based on an attack tree with the AOP and ASP as variables. Section IV presents and analyzes the experimental results obtained and discusses the assumptions and limitations of the study. Finally, Section V provides the conclusions and outlines future research directions.

### Telemedicine system architecture

**Fig. 1:**
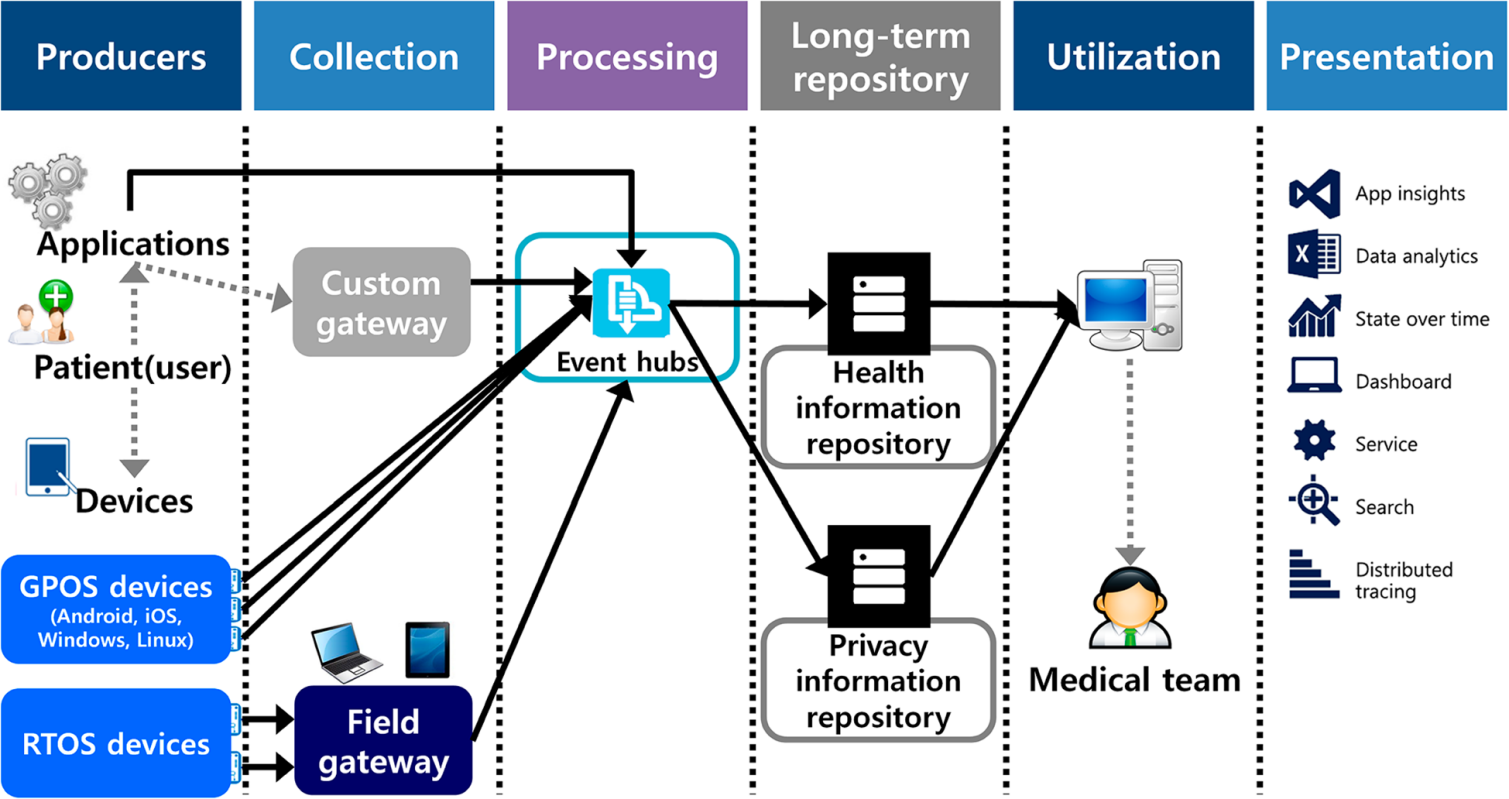
Telemedicine system architecture

A telemedicine system [1] can be divided into two sections according to its components: (1) components accessible to the user (or patient), such as the telemedicine terminal, and (2) components available to the telemedicine service provider only, such as the telemedicine system and medical team. The possible security threat scenarios based on information flow through the various components are summarized below [11, 12] (Fig. 1):

1. Spreading of malicious code in the sensing (measurements) hardware, breaching the security barrier, accessing sensitive patient information, and gaining access to the main server via the sensing device.
2. Information leakage or data forgery in the medical data transmission section.
3. Sensing (measurement) data breach risks due to vulnerabilities in the personal computer (PC), smart device, or gateway used for data transmission by the repository or medical staff.
4. Cyberattack risks due to a vulnerable main server and repository in the provider area.

### Telemedicine system threat extraction and identification

**Fig. 2:**
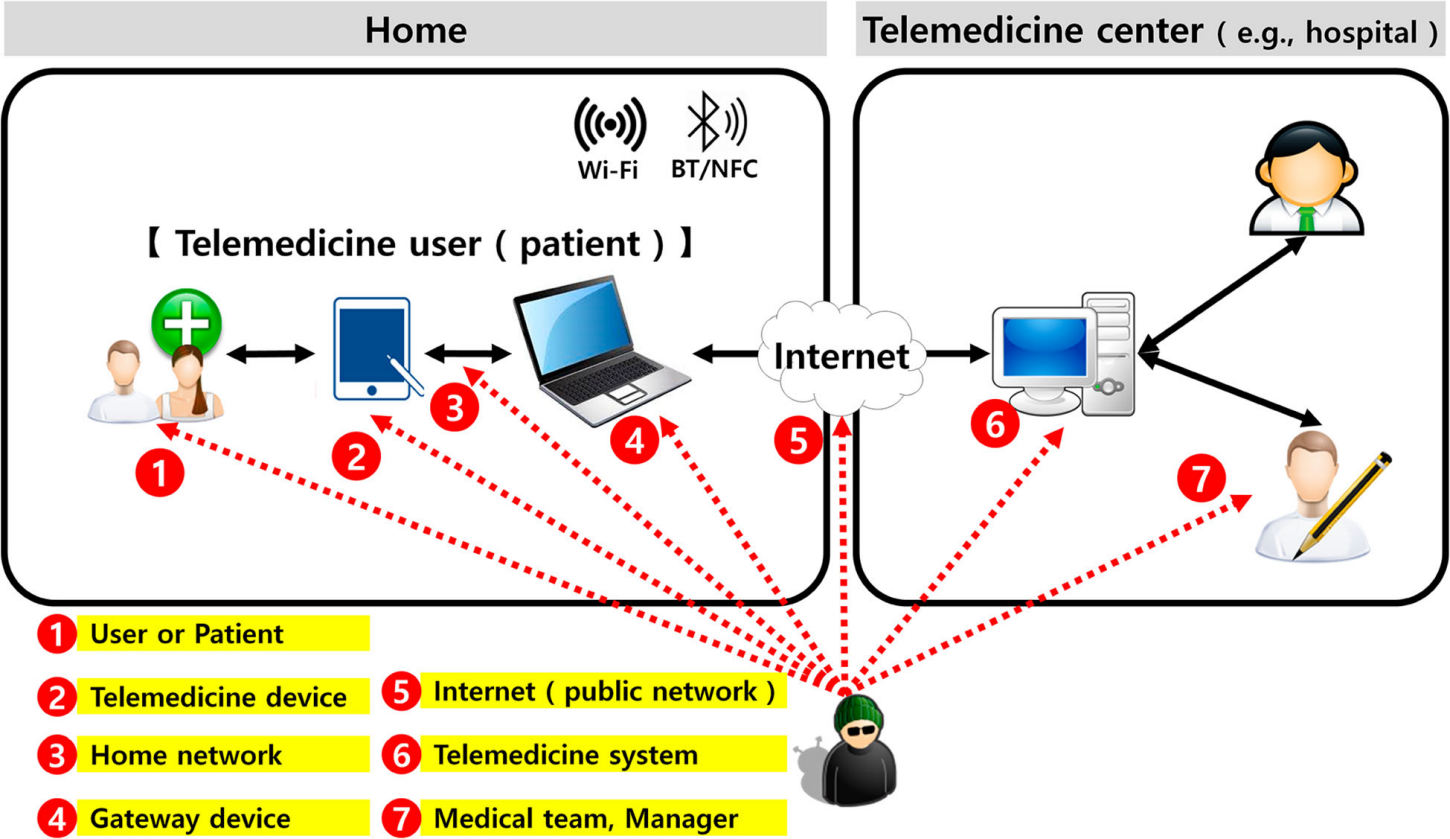
Seven areas related to telemedicine security threats

To identify the threats suitable for constructing the telemedicine attack tree, we extracted typical and scenario-based security threats in accordance with ISO/IEC 27005 Annex C. Examples of typical threats [19] and healthcare-related security threats were extracted based on ISO/IEC 27799 Annex A [44], and the collected data were reorganized. Finally, to identify the telemedicine system vulnerabilities, we reorganized the extracted threats to make them amenable to the telemedicine environment based on ISO/IEC 27005 [19]. The resulting data were used as the components of the telemedicine attack tree. Based on the system architecture and the identified security threats and vulnerabilities, we pinpointed seven telemedicine security threat areas (Fig. 2).

### Use cases: seven telemedicine security threat areas

- Threat #1: User or patient

Users receiving telemedicine (i.e., patients) are most likely residents or senior citizens who live in remote areas. Most of them have never received cybersecurity training and have little interest in cybersecurity. Therefore, their use of telemedicine terminals easily attracts security threats related to device use errors, weak passwords, device loss, phishing, etc. [28].

- Threat #2: Telemedicine devices

A telemedicine terminal is based on either a general-purpose operating system (GPOS) or an embedded-type real-time operating system (RTOS). RTOS-based devices are safe from unauthorized access because they are optimized for specific functions at the design and production stages. Conversely, GPOS-based devices such as smartphones are vulnerable to security threats because they use external apps. The use of telemedicine terminals in such environments makes them vulnerable to security threats owing to the data saving and sharing functionalities of these devices and the risk of device loss/theft, app vulnerabilities, and plaintext transmission [28, 30, 45–47].

- Threat #3: Home network

**Fig. 3:**
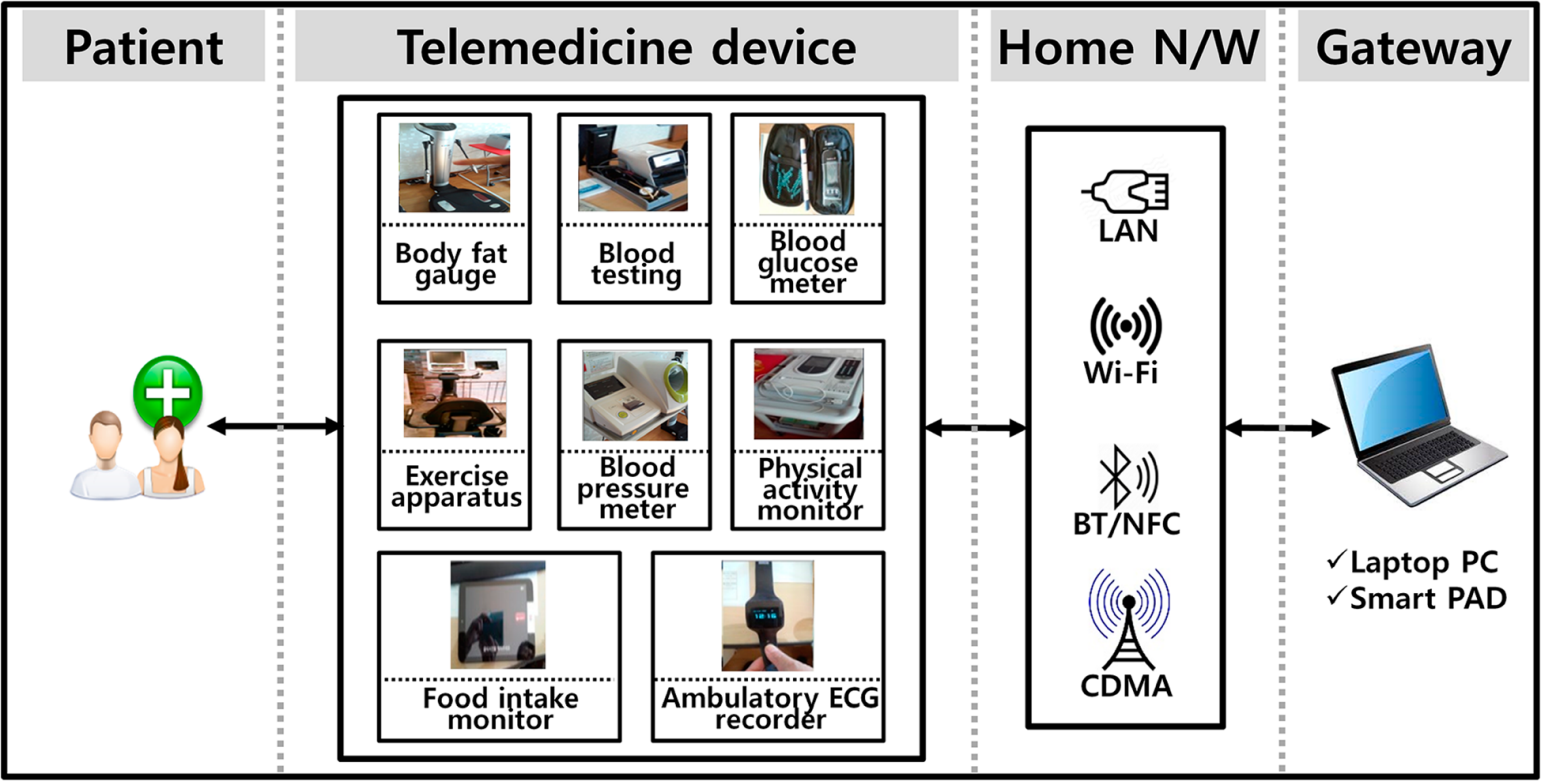
Telemedicine home network

Information transmission between the telemedicine terminal in the private space of the patient (home or office) and the telemedicine system occurs primarily via a wireless network. As illustrated in Fig. 3, the types of networks used in home environments include LAN (local area network), Wi-Fi, Bluetooth, NFC (near field communication), and third and fourth generation/long-term evolution networks. While some embedded-type devices need to be connected to LANs, GPOS-based smart devices can communicate with telemedicine systems via multiple paths. In such environments, home-network-based telemedicine service systems are exposed to security threats associated with end-to-end plaintext transmission and man-in-the-middle (MITM) attacks (Fig. 3) [28, 48].

- Threat #4: Gateway devices

A gateway plays an intermediary role between the patient and telemedicine system, exposing the system to security threats associated with rogue gateways as well as the loss/theft of the gateways and MITM attacks [28, 49].

- Threat #5: Internet (public network)

Communication between the patient and telemedicine system occurs via a public network (the Internet). As private, medical, and health information along with prescriptions are transmitted via the publicly accessible Internet, it is important to establish end-to-end security guidelines. In addition, encrypted data transmission is essential. In this environment, the telemedicine system is vulnerable to security threats associated with sniffing, forgery/alteration, and privilege escalations [28].

- Threat #6: Telemedicine system

The telemedicine system is situated at the location of the telemedicine service provider. It consists of a PC and the software necessary for remote consultations, and its users are the medical staff, nursing personnel, and system administrators (security officer and other support staff). This system is very important because it handles all of the data of the patients receiving the telemedicine services. Moreover, if the telemedicine system is connected to the relevant agencies via the government network hub, stringent security guidelines are necessary to prevent infiltration of the government system. In special cases, telemedicine systems are also used for wireless communication between the exercise equipment used by patients and computers used for remote consultation in telemedicine clinics. In such environments, telemedicine systems can attract security threats associated with MITM attacks, malicious code, telemedicine app forgery/alteration, and illegal network access via physical security checks circumvention [28].

- Threat #7: Telemedicine service provider

**Fig. 4:**
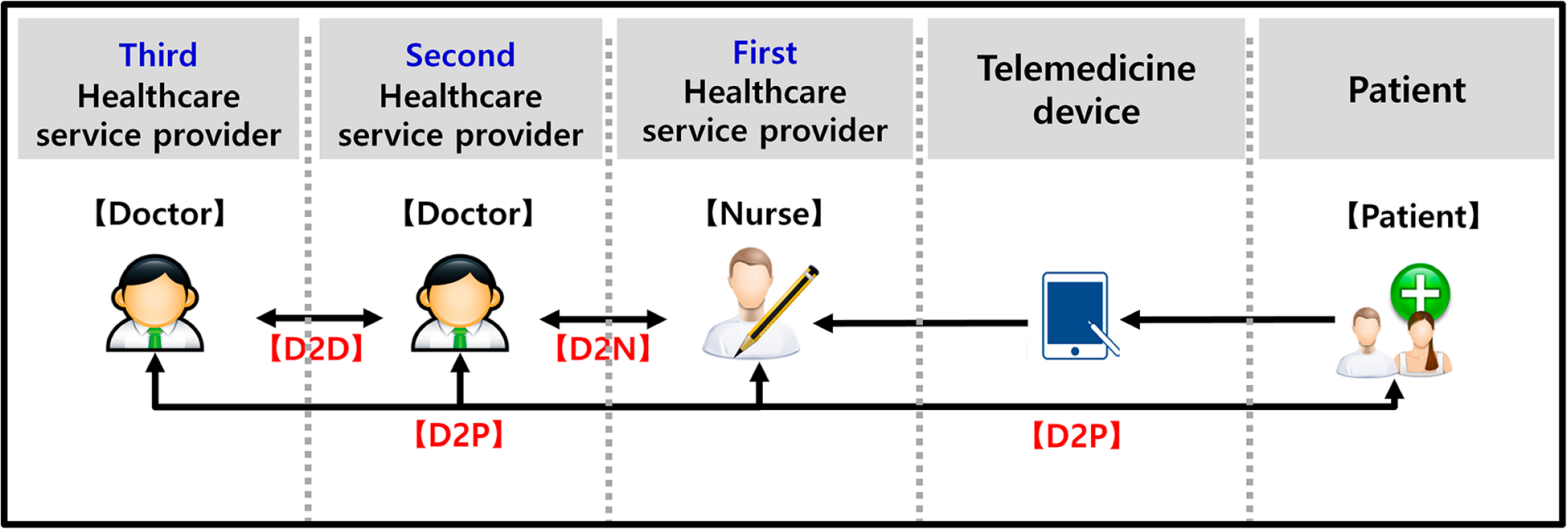
Telemedicine service provider

Telemedicine systems primarily involve doctor-to-doctor (D2D) and doctor-to-patient (D2P) interactions. D2D telemedicine is characterized by the sharing and monitoring of health and medical information and requires higher-level cybersecurity because it involves remote consultation, including the writing of prescriptions. Figure 4 shows a block diagram of D2D and D2P interactions. In this environment, the telemedicine system can attract security threats associated with MITM attacks, malicious code, telemedicine app forgery/alteration, and illegal access of Korea-Net by circumventing the physical security checks present [28]. It can also be vulnerable to security threats associated with device use errors, prescription alterations, leakage of important data, and wiretapping (see Fig. 4).

The security threats likely to be encountered in each of the seven telemedicine service areas above were used as the basic data to calculate the AOP from the attack tree, which was constructed as described in Section III.

## Methods

### Overview

**Fig. 5:**
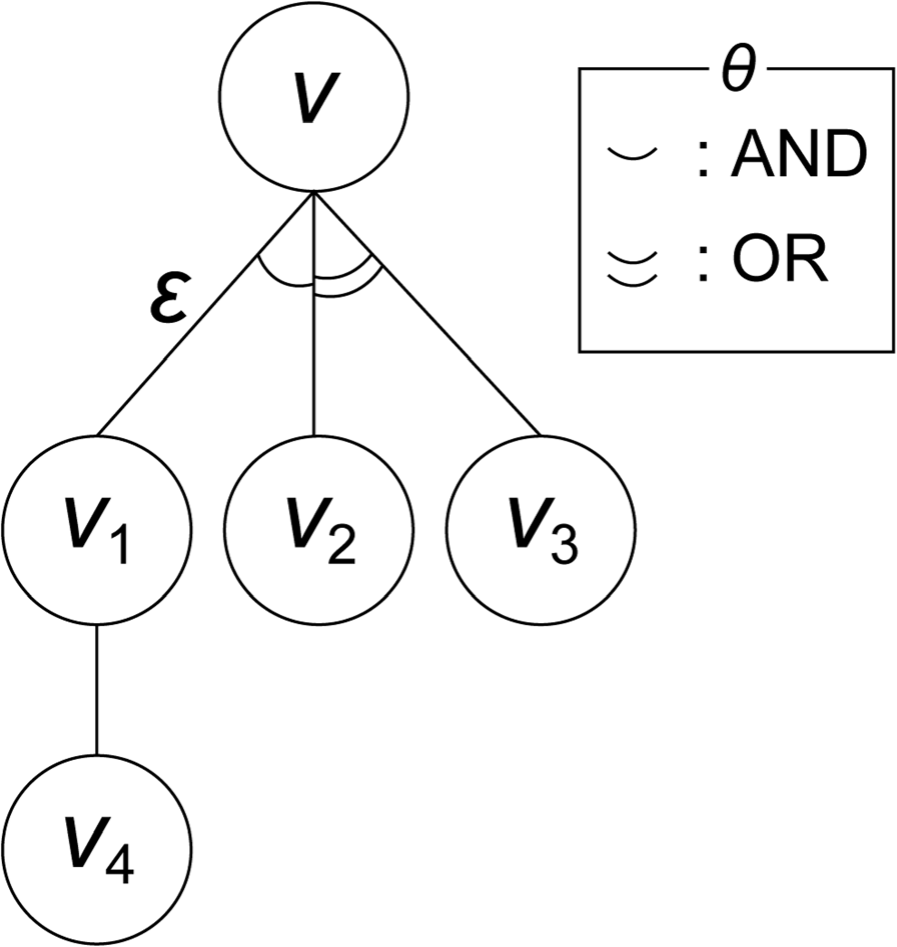
Attack tree

The first step in telemedicine risk assessment is to identify the assets involved and calculate their values. The attack tree is used to estimate all security threats likely faced by each asset, as identified in each of the seven telemedicine security threats areas. As illustrated in Fig. 5, the AOP is calculated using the OR and AND connectors, which are the gates for each node representing attack advancement towards the goal (see Fig. 5).

The main advantage of an attack tree is that it allows defenders to identify potential attacks and appropriate countermeasures. Furthermore, attack trees are originally “self-documented” to facilitate interpretation. The downsides of this approach are that it is difficult to enumerate all of the actions of the attackers and that the expressive power to model attacks that involve simultaneous actions is lacking. In this study, risk assessment methods including ASP and AOP variables were investigated to address these shortcomings [37] and allow more accurate identification of attack methods involving attacker behavior.

In principle, the ASP of a potential attack increases in direct proportion to the motivation of the attacker and in inverse proportion to the effort required for mounting the attack. In this study, the asset value, AOP, and ASP were used as the parameters to assess the security risks associated with telemedicine.

**Fig. 6:**
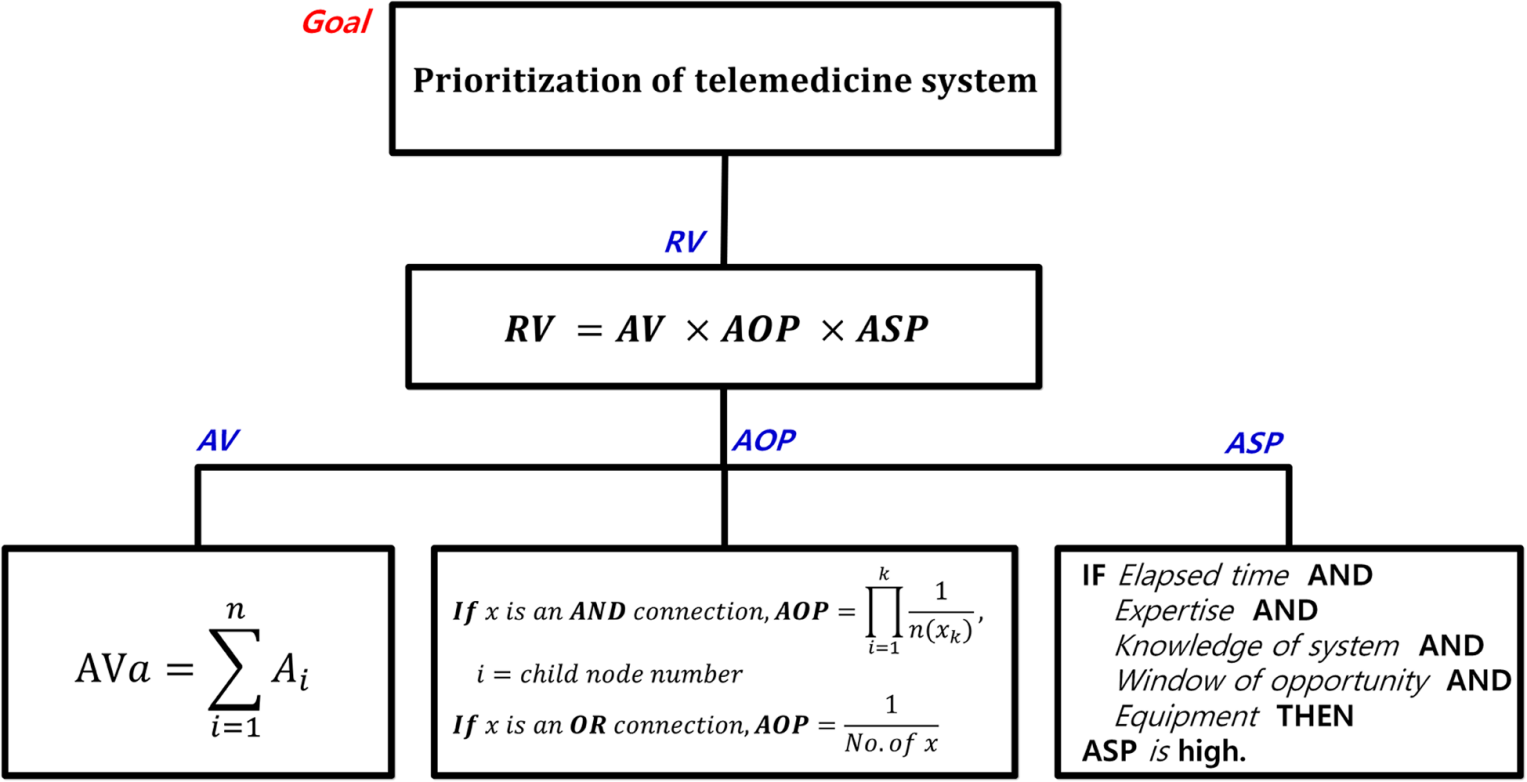
Telemedicine system risk assessment phase

Figure 6 presents an example of how risk assessment is conducted. The risk assessment procedure can be summarized as follows.

1. Evaluate the AV of the telemedicine system (see Tables 1, 2, and 3).
2. Estimate the AOPs of internal and external attacks on the telemedicine system (see Table 4).
3. Estimate the internal and external ASPs of the telemedicine system (see Tables 5, 6, and 7).
4. Select a priority target for security application of the telemedicine system (see Tables 8 and 9).

**Table 1:** Asset value evaluation criteria [19, 44, 49–52]

Division	Low	Moderate	High
Confidentiality	1	2	3
Integrity	1	2	3
Availability	1	2	3
Asset contribution	1	2	3

**Table 2:** Categorization of asset values [19, 44, 49–52]

Security objective	Potential impact	Description
Confidentiality	High	Should be available internally to authorized persons only; unauthorized exposure can result in harm to individual privacy and/or fatal damage to telemedicine system
	Moderate	Can be disclosed internally but in case of external exposure may cause significant problems with respect to individual privacy and/or telemedicine system
	Low	If exposed to external persons, will have negligible effect on individual privacy and telemedicine system
Integrity	High	Accidental or intentional changes may result in extreme harm to individual privacy or telemedicine system
	Moderate	Accidental or intentional changes may cause significant damage to individual privacy or telemedicine system
	Low	Accidental or intentional changes will have negligible effect on individual privacy or telemedicine system
Availability	High	Service interruption may cause fatal damage to operation of telemedicine system
	Moderate	Service interruption may result in significant damage to telemedicine system
	Low	Service interruption will cause negligible damage to telemedicine system
Asset Contribution	High	Asset is essential to telemedicine system services
	Moderate	Asset is partially necessary for telemedicine system services
	Low	Asset plays a supporting role in telemedicine system services

**Table 3:** Definitions of grades for information classification [19, 44, 49–52]

Importance grade	Total score	Description
1	4–5	May cause damage to assets but has almost no influence on telemedicine system
2	6–7	If asset is damaged, has little effect on related domain or system
3	8–9	Asset damage results in significant loss to telemedicine business
4	10–11	Asset damage leads to very significant loss to telemedicine business
5	12	Asset damage leads to very high loss to telemedicine business, which may stop functioning

**Table 4:** AOP evaluation criteria [51, 52]

Division	Low	Moderate	High
	1	2	3
AOP	1–50%	51–80%	81–100%

**Table 5:** Ratings for various aspects of attack potential [51, 52]

Factor	Level	Value
Elapsed time	≤1 day	0
	≤1 week	1
	≤1 month	4
	≤3 months	10
	≤6 months	17
	> 6 months	19
	not practical	∞
Expertise	Layman	0
	Proficient	3
	Expert	6
	Multiple experts	8
Knowledge of system	Public	0
	Restricted	3
	Sensitive	7
	Critical	11
Window of opportunity	Unnecessary/unlimited	0
	Easy	1
	Moderate	4
	Difficult	10
	None	∞
Equipment	Standard	0
	Specialized	4
	Bespoke	7
	Multiple bespoke	9

**Table 6:** ASP ratings [51, 52]

Values	Attack potential required to identify and exploit attack scenario	ASP
0–9	Basic	5
10–13	Enhanced-basic	4
14–19	Moderate	3
20–24	High	2
≥25	Beyond high	1

**Table 7:** Examples of ASP estimates [51, 52]

Attack	Elapsed time	Expertise	Knowledge of system	Window of opportunity	Equipment	Required attack potential	
						Sum	Rating
Leakage of patient information from telemedicine device	0	6	7	4	4	21	High
Forgery via wiretapping and spoofing	0	3	0	4	4	11	Moderate
MITM attacks using rogue AP	0	6	3	10	4	23	High
Health information sniffing	0	0	0	4	4	8	Basic

**Table 8:** RV ratings [51, 52]

Values	Grade
1–12	Low
13–32	Normal
≥33	High

**Table 9:** Examples of telemedicine risk assessment estimates

Asset		AV	Concern	AOP	ASP	RV	
Telemedicine device	RTOS/ GPOS/ gateway	5	Patient information leakage	1	2	10	L
		5	Weak password set	2	5	50	H
		5	Critical information transmitted owing to device operation errors	3	4	60	H
		5	Loss due to improper management of telemedicine device	2	5	50	H
		5	Access to internal system used by unapproved device	1	1	5	L
		5	Information leakage by device because of malware infection	1	1	5	L
		5	Saving important information in device	2	4	40	H
		5	Leakage of significant information from lost/stolen device	2	4	40	H
		5	Access to internal system and disclosure of important information owing to application vulnerabilities of device	2	4	40	H
		5	Device ↔ plaintext transmission between internal system	3	5	75	H
		5	Device ↔ plaintext transmission between telemedicine system	3	5	75	H
		5	Device ↔ MITM attacks between telemedicine system	3	1	15	M
		5	Gateway ↔ plaintext transmission between internal system	3	3	27	M
		5	Information leakage because of malware infection (vaccine or latest patch)	1	2	10	L
		5	Significant information disclosure by gateway hacking	2	1	10	L
		5	MITM attacks using rogue gateway	2	1	10	L
		5	Significant information leakage from lost/stolen gateway device	2	3	30	M
PC	PC	4	Forgery via wiretapping and spoofing	3	5	60	H
		4	Unauthorized access via MITM attacks	2	3	24	M
		4	Gateway ↔ plaintext transmission between telemedicine system	3	5	60	H
		4	MITM attacks using rogue AP	2	1	8	L
		4	Information leakage because of malware infection (vaccine or latest patch)	1	2	8	L
		4	Significant information disclosure owing to gateway hacking	1	1	4	L
		4	Internal access to national communication networks by bypassing physical security controls	1	1	4	L
		4	Internal access to national communication networks by exploiting wireless network vulnerability	1	1	4	L
		4	Leaving working seat for a long period after logging in	2	5	40	H
		4	Nonrepudiation failure by not saving accessed records	1	5	20	M
		4	Accident due to telemedicine system operation errors	1	5	20	M
S/W	Telemedicine software	4	Access to internal system and important information disclosure by exploiting vulnerabilities of application used for telemedicine treatment	1	1	4	L
		4	Access to internal system via update files for application used for telemedicine treatment	1	1	4	L
	Data transmission software	3	Access to internal system and important information disclosure by exploiting vulnerability of application used for data transmission	1	1	3	L
	Patient medical information software	3	Access to internal system via update files for software	2	1	6	L
	Monitoring software	2	Access to internal system via update files for software	2	1	4	L
	ECG software	5	Access to internal system via update files for telemedicine system	2	1	10	L
Information	Personal information	4	Sniffing	3	3	36	H
	Health information	4	Health information sniffing	3	3	36	H
	Medical information	5	Sending invalid prescriptions by changing medical information during telemedicine treatment	1	1	5	L
		5	Misuse of medical information by analyzing network packets during telemedicine treatment	2	1	10	L
		5	Accidents caused by telemedicine system operation errors	2	5	50	H
		5	Forgery via network eavesdropping and spoofing during patient information exchange	2	3	30	H

The procedure enables the actual telemedicine system to identify both hardened targets and targets that require security.

### Asset value

The U.S. National Institute of Standards and Technology (NIST) developed a risk management framework (RMF) to protect computer networks from cyberattacks [53]. The NIST-RMF guidelines categorize risk management activities into the following six security lifecycle steps: (1) categorize, (2) select (based on factors such as minimum security requirements and cost analysis), (3) implement (tailor to the given security environment), (4) assess (determine whether the operation is as intended), (5) authorize (determine whether the risk is acceptable), and (6) monitor (detect changes or signs of attack). Federal Information Processing Standards Publication 199 (FIPS PUB 199) defines the categorization criteria for information and information system security (based on the potential impact of the system) to provide a common framework for taxonomy. It sets three security objectives (confidentiality, integrity, and availability) and defines the levels of the potential effects of security breaches on individuals and organizations as low, moderate, and high [54].

When categorizing threats, the total asset value for each asset to be protected is calculated as follows:

1 $$ \mathrm{AV}a\left( asset\ value\right)={\sum}_{i=1}^n{A}_i, $$

where AV_*a*_ is the sum of the asset values (3–12) of asset *a*, calculated as the sum of the areas associated with the asset values (1–3: contributions of confidentiality, integrity, and availability). Table 1 lists the criteria for asset value evaluation. The asset values of each of the four evaluated items (security objectives) are rated on a three-point scale. The total asset value score is calculated by adding all of the individual scores, and the asset value grade is determined based on the calculated result.

The asset value is assessed in terms of each of the four security objectives (confidentiality, integrity, availability, and asset contribution) at three levels corresponding to the potential effects of each security objective, as described in Table 2, and varies between 3 and 12. By substituting the calculated value into Eq. (1), the asset-value-dependent importance grade, which ranges from 1 to 5, can be obtained.

Table 3 presents the definitions of each of the importance grades categorized above. The evaluated asset values are analyzed using mutatis mutandis, ISO/IEC 27005 [19], and ISO 31000 RM [50] and examined using mutatis mutandis, the risk assessment method based on confidentiality, integrity, and availability, as per NIST 800–37 RMF, FIPS PUB 199, and failure mode, effects, and criticality analysis [55].

### AOP

The AOP is defined as the ratio of the number of attack events of all of the children to the number of attack nodes linked to the parent node in order to achieve the attack goal of the parent node. It is calculated as follows [53]. Let the child node (“X”) be a leaf node; then, AOP = 1 (see Eqs. (2) and (3)).

2 $$ If\ x\  is\ an\ AND\ connection, AOP={\prod}_{i=1}^k\frac{1}{n\left({x}_k\right)},i= child\ node\ number $$

3 $$ If\ x\  is\ an\ OR\ connection,\kern0.5em AOP=\frac{1}{No. of\ x} $$

**Fig. 7:**
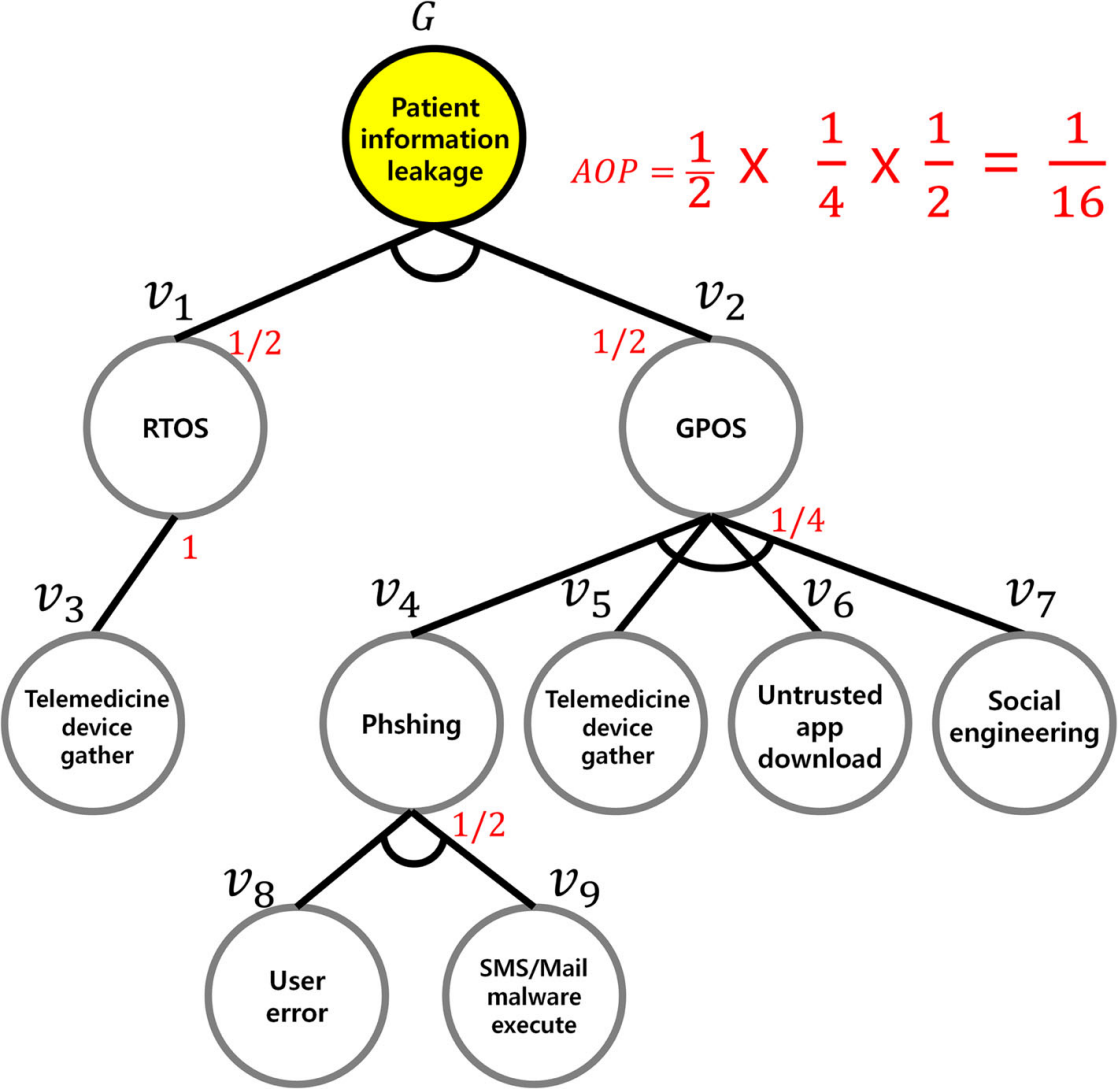
Example of a user or patient attack tree

However, such an attack tree scenario has two major limitations. First, no weight is assigned to the nodes, even though every node has a different risk level and its potential threat can result in different degrees of damage. Second, in lieu of comparison of the node occurrence probabilities, only the probability for achieving the upper node goal is indicated without considering the node occurrence frequency and risk level of each node, making it difficult to quantify the security threat vulnerabilities of telemedicine devices. The AOP is calculated by designing an attack tree for each security threat scenario according to the seven telemedicine security threats areas, as illustrated in Fig. 7.

The AOP for the example in Fig. 7 can be calculated as follows. Because ν_8_ or ν_9_ can be selected to move to ν_4_, ν_2_ has an AOP of 1/2. Further, as one of the methods represented by ν_4_, ν_5_, ν_6_, and ν_7_ must be selected to achieve ν_4_, its AOP is 1/4. Because the single node ν_3_ is selected to achieve ν_1_, its AOP is 1. Consequently, if the attack target is the user, the AOP for patient information leakage is calculated to be 6.25%, as follows:

4 $$ AOP=\frac{1}{2}\times \frac{1}{4}\times \frac{1}{2}=\frac{1}{16}\times 100. $$

Following attack tree construction for each of the seven telemedicine security threat areas, the AOP of each attack tree is calculated, and a score assigned to each area accordingly. An AOP assessment grade is allocated to each area based on a three-point scale, as per the AOP value calculated by Eq. (4) and in keeping with the evaluation criteria (Table 4).

### Asp

The ASP, defined in ISO/IEC 15408 [51] and ISO/IEC 18045 [52], is assessed based on the following factors [52]:

- Time taken by an attacker to identify a vulnerability, develop an attack method, and mount the attack
- Specialist expertise required
- Knowledge of the system under investigation
- Window of opportunity to access the attack target
- IT hardware/software or other equipment required to identify and exploit a vulnerability

These factors affecting the ASP are not independent, but rather are interchangeable from various angles. For example, the expertise and equipment needed can be replaced by the elapsed time (see Table 5).

The ASP is calculated by applying the factor value (Table 5) as per the attack scenario for the seven telemedicine security threat areas. Subsequently, a rating is assigned based on the attack potential value (see Table 6), and categorization is performed based on the attack potential level (see Table 7). To calculate the ASP of each security threat, the categorized ASP levels are mapped onto the leaf nodes of the attack tree. For example, each leaf node in Fig. 7 is mapped at the ASP level assigned to it according to the ASP estimates (see Table 7).

### Risk

The telemedicine risk value (RV) is the product of the AV, AOP, and ASP:

5 $$ RV=\mathrm{AV}\times \mathrm{AOP}\times \mathrm{ASP} $$

The calculated RVs are assessed at three levels: low, normal, and high (see Table 8).

**Fig. 8:**
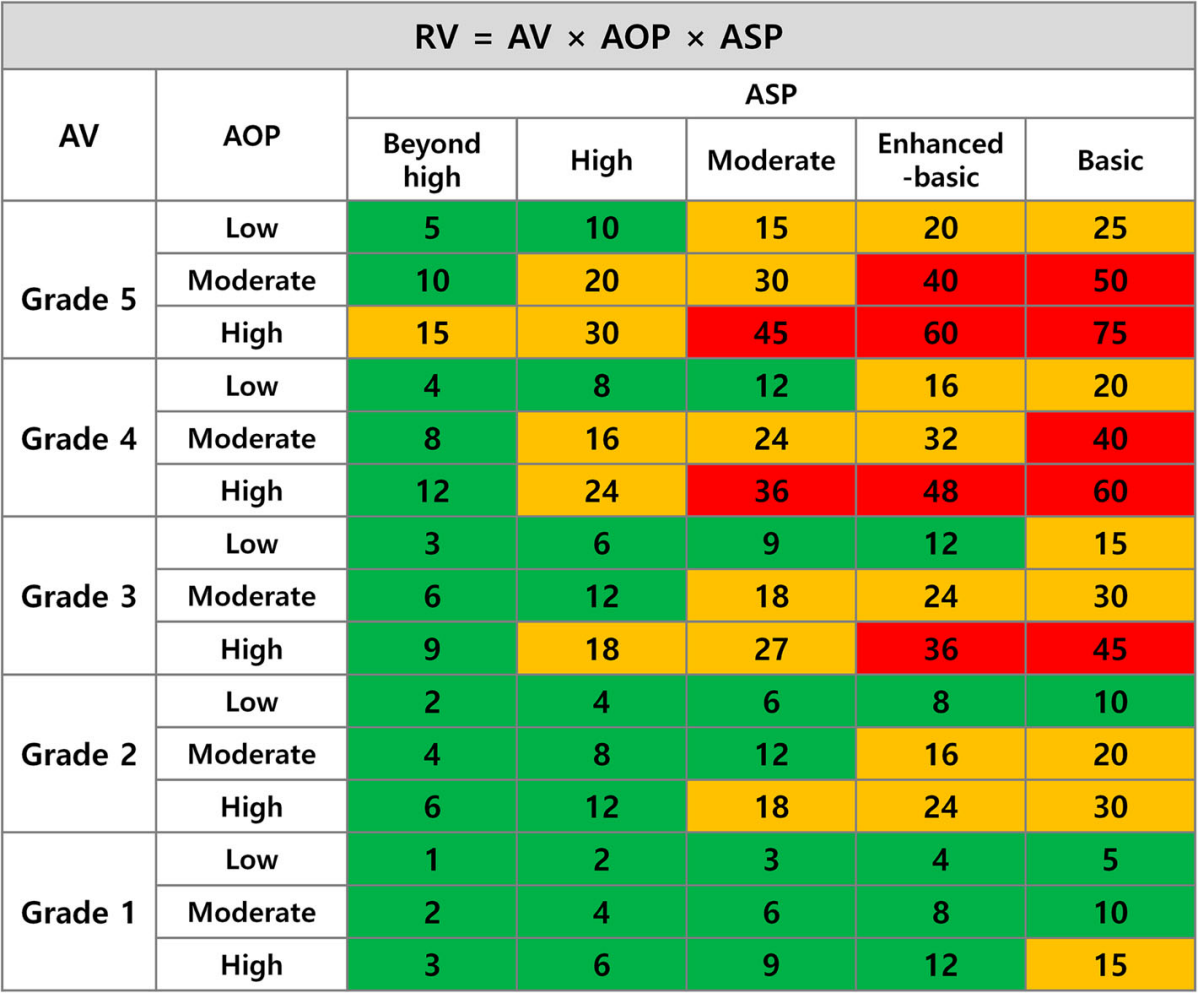
Examples of RV estimates

When interpreting the risk assessment results, the higher the AV, AOP, and ASP, the higher the RV (see Fig. 8).

## Results

The telemedicine risk analysis results represent the security threat risk levels and can be interpreted in terms of the relative effect of a given attack. It is necessary to establish the appropriate security guidelines based on the AV of each threat while considering its AOP and ASP (see Table 9).

In this study, the most popular modelling method, an attack tree, was applied to the telemedicine environment, and the security concerns for telemedicine systems were found to be very large. Risk management and evaluation methods suitable for the telemedicine environment were identified, and their benefits and potential limitations were assessed.

## Discussion

In this study, data were collected via on-site verification and security vulnerability analysis (intrusion testing, threat modeling) of the telemedicine system shown in Table 7, and models were analyzed based on assumptions. Table 1 lists the three-point classification approach employed based on the RMF [19, 44, 49–52]; in addition, the importance of the telemedicine system can be evaluated by referring to Tables 2 and 3. The proposed model uses attack tree modeling to evaluate the ASP and AOP to estimate the total risks of remote healthcare systems, accounting for security threats. This report provides a method of evaluating cybersecurity risks in remote medical systems, an area of technological convergence for recently illuminated untact (i.e., non-face-to-face) [56] medical services.

The limits of the proposed model are that the technical environment of the hospital should be considered when applying the model to the telemedicine system and the participation of telemedicine professionals is necessary. Another limitation is that biomedical engineers may not always be able to accept the outcome of security threat prioritization, and the weight of each criterion and/or the severity of the assigned security grade may have to be reassessed and reassigned. The analysis of security threats in a telemedicine environment requires the participation of information security experts with medical expertise and the cooperation of medical professionals. Such analyses can be performed using methods such as those employed to intelligently analyze forecasting data mining techniques. Intelligent analysis of prediction data mining techniques is widely used to support optimization of future decision-making in various fields, including healthcare and medical diagnoses. The methods used include Chi-squared Automatic Interaction Detection (CHAID), Exchange Chi-squared Automatic Interaction Detection (ECHAID), Random Forest Regression and Classification (RFRC), Multivariate Adaptive Regression Splines (MARS), and Boosted Tree Classifiers and Regression (BTCR) [57–64].

Nevertheless, this research will contribute significantly to the literature by facilitating the assessment and prioritization of cybersecurity risk factors lacking prior research in the telemedicine sector.

In addition, at a time when the need for noncontact medical care is growing due to concerns about infectious diseases such as CoV, countermeasures against new security threats resulting from the convergence of ICT with the medical sector, such as through telemedicine and precision medicine, are essential.

## Conclusions

The range of cybersecurity problems associated with telemedicine services necessitates the implementation of security guidelines for the maintenance and management of appropriate security measures that address the security threats posed to each of the seven areas of telemedicine services identified in this paper. The results of the security threat assessment and analysis performed in this study should serve as the basis for establishing efficient security guidelines in telemedicine environments. In the current healthcare service environment, wherein telemedicine services are provided by outsourced ICT personnel without medical security backgrounds, telemedicine is highly prone to cyberattacks.

There is a huge risk that life could be affected if a cyberattack modifies information that is normally prescribed for telemedicine services. Thus, telemedicine is a very important system that must be considered for safety as well as security. By presenting a systematic approach for security threat identification and vulnerability diagnosis, this study will further telemedicine usage while ensuring its safe and smooth operation.

In a follow-up study, the AOP values estimated in this study will be verified through mockup tests performed in real-life settings, and a process or security verification algorithm will be developed to counter the security threats faced based on prioritization of the security requirements determined from the risk assessment performed. Additionally, the concept of “precision medicine” has led to a personally customized medical era and the application of optimized diagnosis and treatment based on personal health information such as genetics and lifestyle information. Further research will be required to address the ever-increasing number of cybersecurity threats in the medical paradigm as ICT and medical technologies evolve.

This paper provides a method of attack tree modeling and analysis for cyber risk management. The basic elements of this modeling approach were reviewed, and the limitations of the approach were discussed. In future research, additional cyber risk modeling paradigms will be investigated, such as binary decision-making diagrams and Markov models, to identify the limitations of their representativeness and their abilities to quantify and mitigate risks. In addition, research on ways to identify and mitigate new security threats to telemedicine will be needed, as the need for untact (i.e., non-face-to-face) [56] medical services increase due to issues related to infectious diseases such as CoV. Theoretical generalizations for these mathematical modeling techniques will then be developed to overcome these limitations.

## Data Availability

All data generated or analyzed during this study are included in this published article.

## Abbreviations

ICT: Information and communication technology
PACS: Picture Archiving Communications System
IMD: Implantable medical device
PC: Personal computer
RTOS: Real-time operating system
GPOS: General-purpose operating system
MITM: Man-in-the-middle
D2D: Doctor-to-doctor
D2P: Doctor-to-patient
ASP: Attack success probability
NIST: National Institute of Standards and Technology
RMF: Risk management framework
FIPS PUB 199: Federal Information Processing Standards Publication 199
AOP: Attack occurrence probability
RV: Risk value
